# P17 Infectious sacroiliitis: an uncommon septic arthritis

**DOI:** 10.1093/rap/rkae117.048

**Published:** 2024-11-05

**Authors:** Su Aung, Sai Sai, Robert Wekesa

**Affiliations:** Lincoln County Hospital, Lincoln, United Kingdom; Lincoln County Hospital, Lincoln, United Kingdom; Lincoln County Hospital, Lincoln, United Kingdom

## Abstract

**Introduction:**

Infectious sacroiliitis, an infection of the sacroiliac joint, can cause severe morbidity if not promptly treated. *Staphylococcus aureus* is a common pathogen, especially problematic in patients on chronic hydrocortisone therapy due to its immunosuppressive effects. This case report discusses the diagnosis and treatment challenges of infectious sacroiliitis in such immunocompromised patients, highlighting the necessity of a minimum six-week targeted antibiotic regimen to ensure bacterial eradication and prevent relapse. The report emphasises aggressive and sustained treatment for optimal outcomes in these vulnerable patients.

**Case description:**

A 47-year-old man was admitted with fever, constant feeling of tiredness, and severe right-sided gluteal pain radiating to the right lower extremity, worsening with movement. He has a history of ACTH deficiency, managed with 25 mg of oral hydrocortisone daily. Examination revealed skin eczema, tenderness in the right gluteal region, and an inability to flex the right hip. His temperature was 39 °C, and CRP levels were over 400 mg/L (normal < 5 mg/L). Two sets of blood cultures showed *Staphylococcus aureus*. Echocardiogram ruled out of infective endocarditis.

An MRI of the lumbar spine showed edema in the right posterior paraspinal muscles at L4, while a CT scan of the abdomen and pelvis revealed fat stranding around the second sacral vertebra and significant inflammation in the right iliacus and distal psoas muscles. The patient was treated with 21 days of intravenous flucloxacillin, resulting in negative blood cultures and improved inflammatory markers, leading to discharge.

One week later, he was readmitted with severe gluteal pain and fever. Blood cultures again showed *Staphylococcus aureus*. A repeat MRI indicated an abnormal signal around the right sacroiliac (SI) joint, with extensive edema and swelling in the right gluteal, iliacus, posterior paraspinal, and obturator muscles, suggesting sacroiliitis. The patient received six weeks of intravenous flucloxacillin. After this extended antibiotic therapy, his pain and fever resolved, laboratory parameters normalised, and blood cultures were negative.

**Discussion:**

Bacterial infection is a serious cause of arthritis, with infective sacroiliitis being particularly rare, accounting for just 1-2% of all septic arthritis cases. Diagnosing this condition can be challenging despite clear symptoms, often leading to delays.

This case highlights several important aspects of managing severe musculoskeletal infections. Firstly, it emphasises the need for a comprehensive diagnostic approach, utilising blood cultures, imaging, and echocardiography to precisely identify the source and extent of the infection. Treating deep-seated musculoskeletal infections is especially challenging in patients with underlying conditions which can complicate the clinical picture and impact immune response.

The recurrence of infection despite initial appropriate antibiotic therapy suggests the need for prolonged and potentially more aggressive antimicrobial treatment. Close monitoring for complications or recurrence is essential. The decision to restart IV flucloxacillin and extend the antibiotic course to at least six weeks represents a strategic adjustment based on clinical progression and imaging results. Given that the patient is currently on standard hydrocortisone therapy for ACTH deficiency, transitioning to modified-release hydrocortisone therapy may be a better option as it more closely mimics the circadian rhythm of glucocorticoids, normalises the immune cell profile, and reduces the incidence of recurrent infections.

This case underscores the necessity for clinicians to remain vigilant for recurrent infections, considering extended or repeated imaging and microbiological studies in patients not responding as expected to initial therapy. It also highlights the importance of individualised treatment plans, especially for patients with complex medical histories and comorbidities, to achieve optimal clinical outcomes.

**Key learning points:**

• **Diagnostic challenges:** Infectious sacroiliitis is rare and difficult to diagnose, requiring comprehensive approaches including blood cultures, imaging and echocardiography.

• **Immunocompromised patients:** Patients on chronic hydrocortisone therapy are at higher risk for infections like sacroiliitis due to immunosuppression. Modified hydrocortisone may represent a superior option, as it more closely mimics the physiological circadian rhythm of glucocorticoids, normalises the immune cell profile, and reduces the incidence of recurrent infections.

• **Rare disease:** Infectious sacroiliitis is a rare and often overlooked condition that requires a high index of suspicion for accurate diagnosis. Early recognition and prompt treatment are crucial to prevent serious complications and improve patient outcomes.

• **Prolonged treatment:** Initial antibiotic therapy may lead to temporary improvement, but extended antimicrobial regimens (at least six weeks) are often necessary to prevent relapse.

• **Relapse management:** The recurrent of symptoms and positive cultures necessitates vigilant monitoring and possibly more aggressive and prolonged antibiotic treatment.

• **Individualised care:** Effective management of deep-seated infections in patients with complex medical histories requires tailored treatment plans and ongoing assessment to achieve optimal outcomes.

